# Ocular pathology associated with obstructive sleep apnea syndrome

**Published:** 2020

**Authors:** Teodor Răzvan Cristescu, Florin Dumitru Mihălțan

**Affiliations:** * Department of Ophthalmology, Slobozia County Emergency Hospital, Romania; ** “Marius Nasta” Pneumophtisiology Institute, Bucharest, Romania; “Carol Davila” University of Medicine and Pharmacy, Bucharest, Romania

**Keywords:** obstructive sleep apnea syndrome, intraocular pressure, lacrimal secretion, apnea/ hypopnea index

## Abstract

**Objective:** The purpose of this study was to describe the ophthalmological findings in patients with obstructive sleep apnea syndrome (OSAS).

**Methods:** The study group was made up of 65 patients with OSAS diagnosis and the findings were compared with the ones of the control group (n=39), which comprised patients without OSAS. An observational, analytic study, with a transversal component and a prospective component, was performed.

**Results:** The IOP (intraocular pressure) was similar in both groups (p=0,9786). In the OSAS group, IOP increased with a higher AHI (apnea/ hypopnea index), but not significantly (p=0,057). Similarly, there was no correlation between a higher AHI and the lacrimal secretion in the OSAS group (p=0,3282). However, when we compared control with OSAS patients, we found a significantly higher degree of lacrimal hyposecretion in the latter (p=0,0003). CPAP treatment had no effect on IOP, as initial and final medium IOP were 15.06 and 14.89 respectively (p=0,8327). Regarding lacrimal secretion, it seems that CPAP treatment had an improving effect, as tear production rose from a 9.25 mm medium to 10.45 mm on the Schirmer I test (p=0,0118).

**Conclusions:** Overall, OSAS patients showed similar IOP and glaucoma prevalence as the normal population. However, they had an abnormal tear secretion and a higher prevalence of eyelid laxity (a sign of floppy eyelid syndrome).

**Abbreviations**:

OSAS/ SASO = obstructive sleep apnea syndrome, AHI = apnea/ hypopnea index, CPAP = continuous positive airway pressure, FES = floppy eyelid syndrome, TSch/ TSCH = Schirmer test, IOP = intra-ocular pressure, PSG = polysomnography

## Introduction

The obstructive sleep apnea syndrome (OSAS) is characterized by the narrowing and collapse of the superior airways, which leads to episodes of apnea and sleep deprivation during the night time. On the long run, these episodes have a negative effect on the general health. We can see consequences on many organs and systems of the human body, including the eye [**1**]. Sometimes, patients seek help from a variety of different physicians, and, their background sleep apnea syndrome, which is best diagnosed by a pneumologist or sleep specialist, passes undetected. According to literature, there are many ocular ailments that are associated with OSAS: ectropion, blepharochalasis, blepharoptosis, eyelash ptosis [**2**], floppy eyelid syndrome (FES) [**3**], lacrimal gland ptosis, papillary conjunctivitis, filamentary keratitis, corneal erosions, keratoconus, glaucoma [**4**-**6**,**8**], optic neuropathy [**7**] and papilledema [**9**,**10**].

The method considered as gold-standard for diagnosing and measuring the severity of OSAS is polysomnography (PSG). The main outcome of this diagnostic tool is the apnea-hypopnea index (AHI), which quantifies the number of apneas (stoppages of breathing) and hypopneas (periods of diminished airflow associated with oxyhemoglobin desaturation or with sleep interruption) lasting more than 10 seconds that happen in an hour of sleep. The main current treatment for OSAS is continuous positive airway pressure (CPAP) applied through a nose mask during sleep.

## Objectives

The study had the following two objectives:

1. to establish a connection between sleep apnea syndrome and ocular problems such as glaucoma, high intraocular pressure, dry eye syndrome, floppy eyelid syndrome and others, as literature and studies to this date do not present conclusive evidence.

2. to establish the nature by which OSAS treatment by continuous positive airway pressure (CPAP) modifies or influences these ocular problems.

## Hypothesis

Based on preliminary research and references study, two hypotheses were formulated:

1. the frequency of the ocular afflictions mentioned earlier was different in OSAS patients compared to people from the general population.

2. intra-ocular pressure (IOP), dry eye syndrome and other observed ocular problems improved during CPAP treatment.

## Materials and methods

An observational, analytic study, depending on data collection, which had two components, was performed:

- a transverse component, with data collection at moment 0, making a “picture” of the current medical situation. 

- a longitudinal, prospective component, in which we evaluated pathology evolution after or without treatment, compared to the onset situation.

65 patients with OSAS, hospitalized in the Pneumology Department of “Marius Nasta” Hospital Bucharest, who underwent polysomnography (PSG) studies, were included. Not all the patients were included in all statistics, as data was incomplete for some of them. Patients had an apnea/ hypopnea index above 5 events/ hour of sleep (AHI > 5), both sexes were included, and ages ranged from 34 to 98 years. As far as symptoms are concerned, patients mainly complained of excessive daytime sleepiness and loud snoring, and some also had morning headaches and difficulty in concentrating during the day.

Before night-time PSG, patients were subjected to an extensive eye control, including anterior pole examination, ophthalmoscopy (funduscopy), IOP measurement (portable iCare device) and lacrimal secretion measurement (Schirmer I test - without topical anesthesia). The Schirmer test was stopped when the paper filter reached the 15 mm mark, even if this happened before 5 minutes. Afterwards, any potential correlation with the polysomnography result was analyzed. 

OSAS severity was graded in the three classical categories: 

- mild: AHI between 5 and 15;

- moderate: AHI between 15 and 30;

- severe: AHI over 30 events/ h.

The control group was made up of 39 patients without specific symptoms of OSAS. They were considered as having low risk for developing OSAS, following evaluation with the stop-bang questionnaire (0-2 points) (**Table 1**) and the Epworth sleepiness scale.

**Table 1:** STOP-BANG questionnaire. One point for each “yes” answer

STOP	**S**nore loudly?	yes	no
	**T**ired during daytime?	yes	no
	**O**bserved by others to stop breathing during sleep?	yes	no
	high blood **P**ressure?	yes	no
BANG	**B**MI > 35	yes	no
	**A**ge > 50	yes	no
	**N**eck circumference > 40 cm	yes	no
	male **G**ender	yes	no

Moreover, we looked for any sign of glaucoma and floppy eyelid syndrome in OSAS patients, as according to other studies and the medical literature on this subject, they seemed to have a higher incidence in these patients. Specifically, we paid attention to the optic nerve head appearance, in addition to IOP measurement. We considered Floppy eyelid syndrome as present when superior eyelid laxity was associated with papillary conjunctivitis.

Some of the patients were enrolled in a second part of the study and underwent treatment for OSAS by CPAP. This is the most effective treatment known to this day for sleep apnea, and reverses the airway obstruction during night-time [**11**]. Another group was also followed, which was made up of patients who were not subjected to the CPAP treatment for various reasons (refused treatment, were uncompliant or it was not recommended by the physician), and this was the control group, used for statistical comparison.

The statistical analysis was realized with graph pad prism 8.0. The chi-square test or Fischer’s exact test were used for comparisons of qualitative data. Results of all statistical analyses were evaluated within a 95% confidence interval and the p values less than 0.05 were accepted as significant. To test the difference in the means of the two groups, normals and OSAS groups, we used the T-test, as we assumed the variances of the two populations to be equal. Anova was necessary to compare multiple groups. Correlation analysis and simple linear regressions were used to detect associations between variables.

## Results

**Fig. 1:**
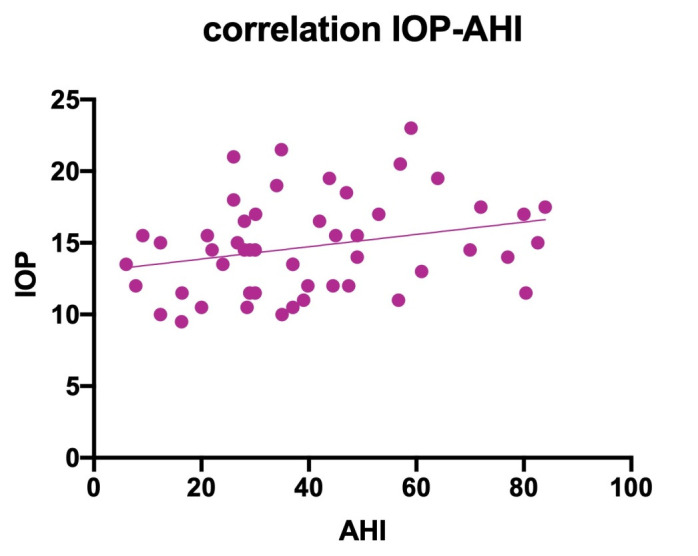
Correlation between the apnea/ hypopnea index and the intra-ocular pressure

**Fig. 2:**
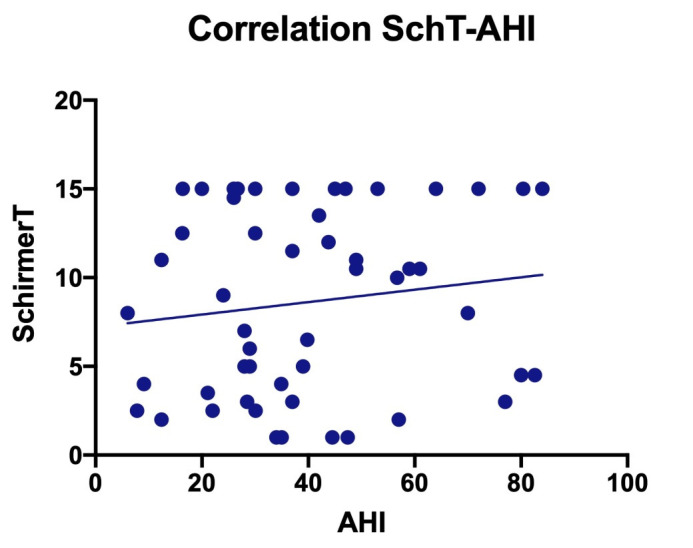
Correlation between the apnea/ hypopnea index and the Schirmer test I results

**Fig. 3:**
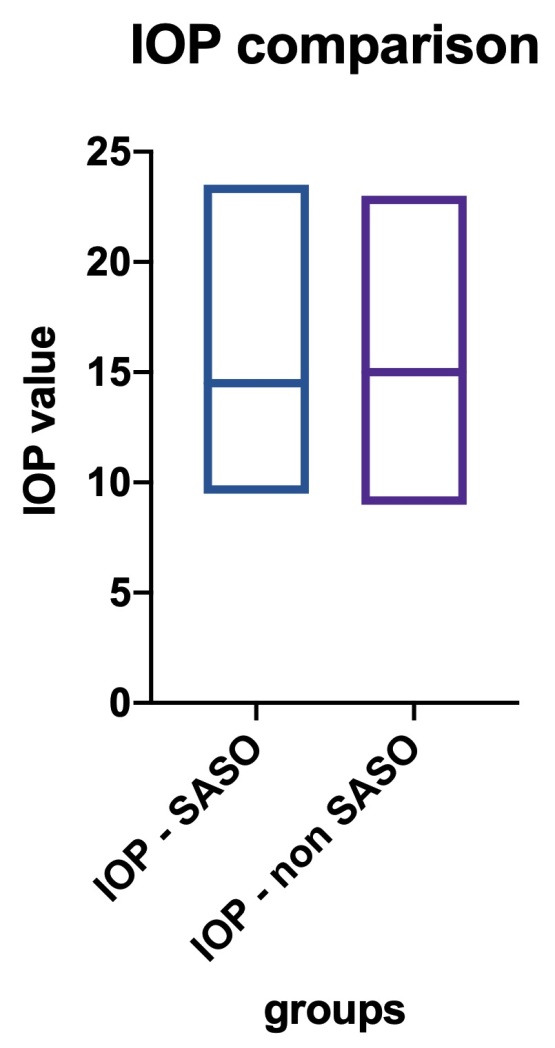
Comparison of the intra-ocular pressure between the study and control groups

**Fig. 4:**
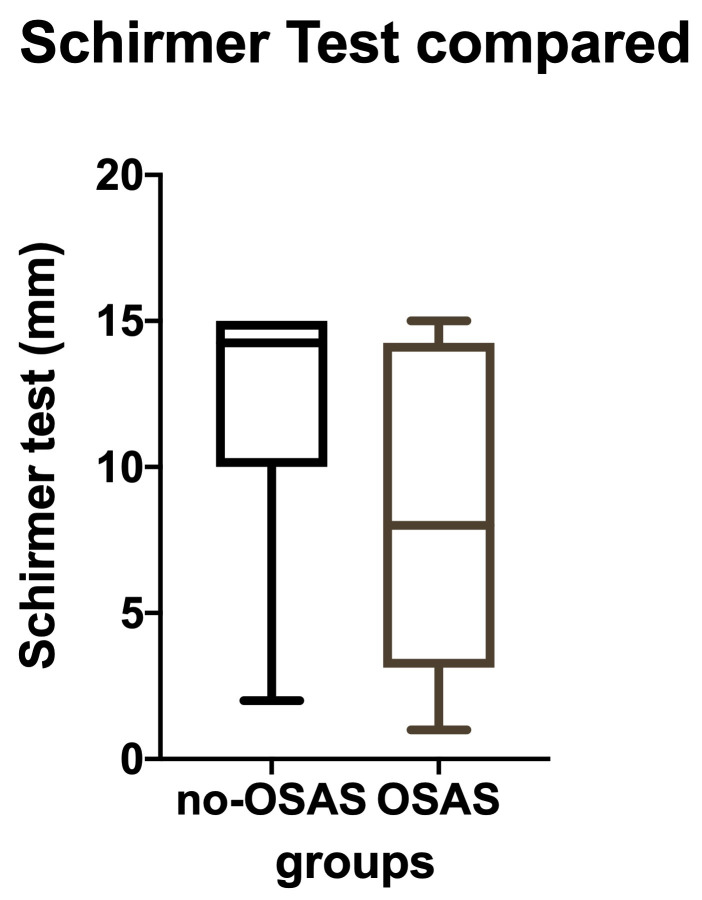
Schirmer test results compared: the study and the control groups

**Fig. 5:**
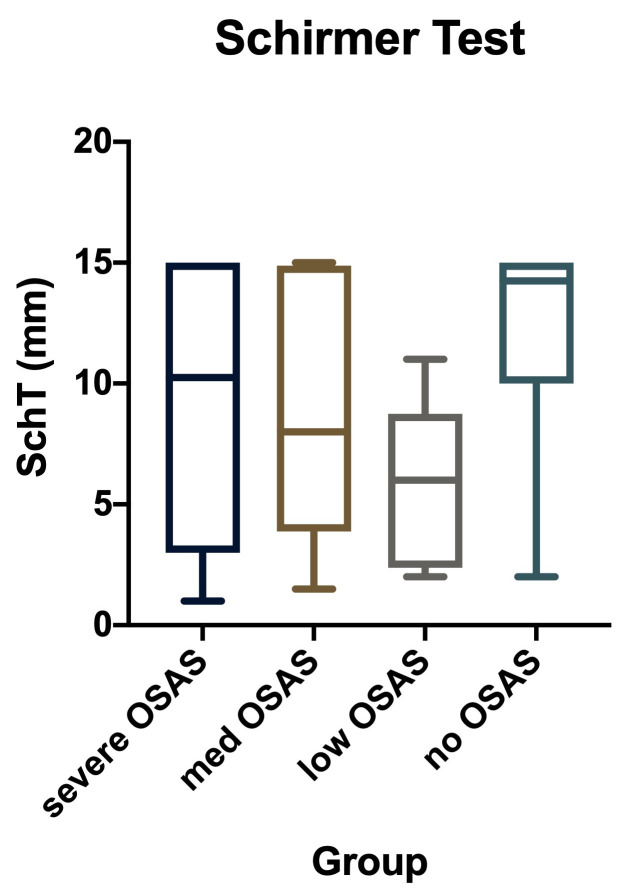
Schirmer test results, comparison of the different severity groups of OSAS

**Fig. 6:**
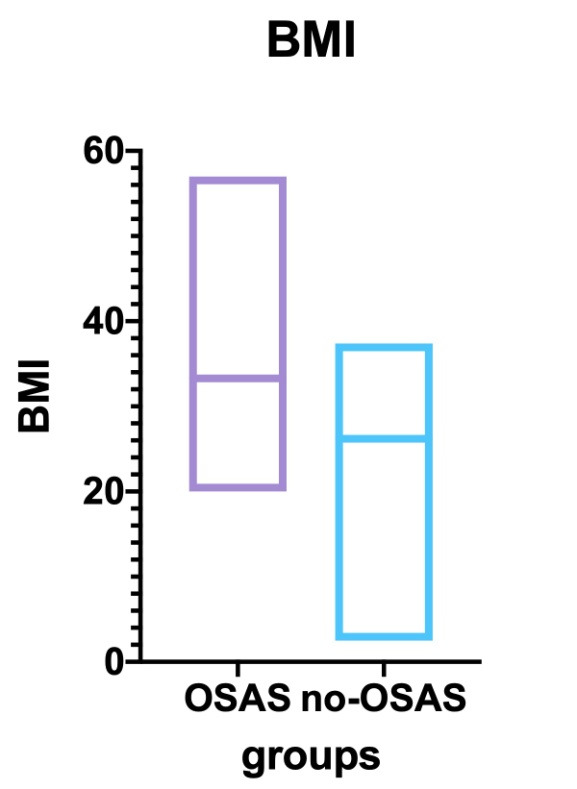
Body mass index compared in the study and the control groups

**Fig. 7:**
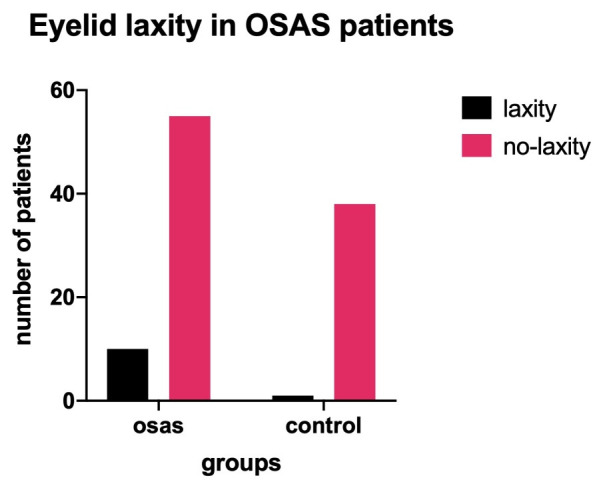
Number of patients with eyelid laxity in the OSAS and control groups

**Fig. 8:**
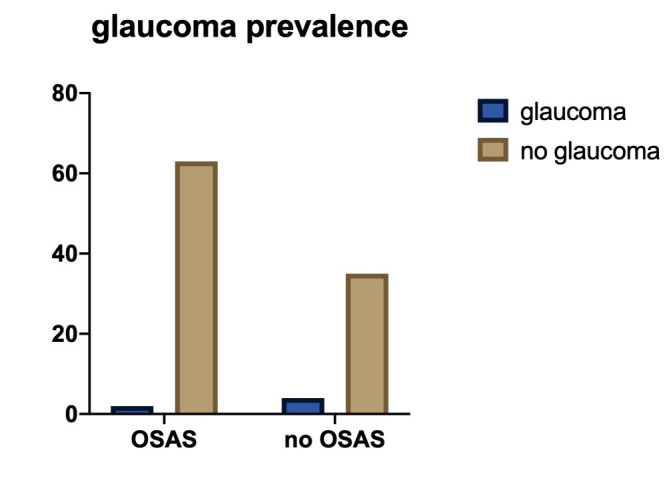
Number of patients with glaucoma in the OSAS and control groups

**Fig. 9:**
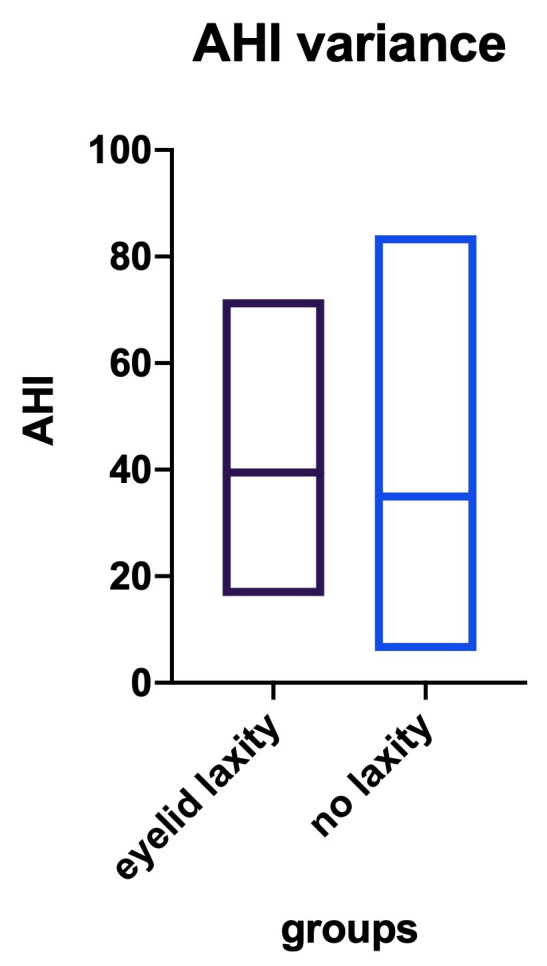
Apnea/ hypopnea index values in patients with eyelid laxity compared with patients without eyelid laxity

• We searched for a connection between intra-ocular pressure and the apnea/ hypopnea index. The result of the simple linear regression analysis showed a tendency of higher IOP in patients with high AHI (**Fig. 1**), but lacked the statistical significance to draw a clear conclusion (p-value: 0,057);

• There was no correlation between lacrimal secretion and AHI. According to the statistical analysis by simple linear regression, we could draw a line with a slope that was not significantly non-zero (r squared = 0,01952, p-value = 0,3282). The AHI explained only 1,952% of the Schirmer test variation (**Fig. 2**).

• We analyzed the relationship between lacrimal secretion (measured through the Schirmer Test) and both the BMI and AHI combined. The multiple linear regression showed the former not to be influenced significantly by the latter two combined (p=0,2599, r squared = 0,045);

• Similarly, the intraocular pressure seemed not to vary function to BMI and AHI combined. (p=0,078, r squared = 0,11), although there was a slight tendency for the IOP to be higher as the latter two worsened.

• We analyzed the differences between intra-ocular pressure in patients diagnosed with OSAS and a control group. In the first group I, 53 patients were included, whilst in the second group, 34 were included (**Fig. 3**). None of the patients in the groups had a diagnosis of glaucoma, as they were previously excluded from this statistic, and none were administered any IOP lowering medication. The values were not statically different, on the contrary, the similarity was high, given a medium IOP of 14.75 mmHg in the first group (SASO group) and 14.76 in the control group (T-test: p-value=0,9786);

• We studied total lacrimal secretion (basal and reflex) for 36 patients without SASO and 52 with the disease. We measured on the Schirmer test and found that SASO patients had lacrimal hyposecretion. Their medium value on the filter paper was 8,35 mm, compared to 12,18 for the normal control group (**Fig. 4**), and the difference was highly significant (T-test: p-value = 0,0003). The same tendency was noticed when we separately analyzed the different severity OSAS groups (one-way ANOVA: p<0,05) (**Fig. 5**).

• The BMI analysis between the two groups (OSAS vs. control) revealed an important difference, with strong statistical significance (T-test: p<0,0001). We compared 47 patients with SASO vs. 34 patients without SASO. The medium BMI for the first ones was 33.52, compared to only 26.57 in the second group (**Fig. 6**).

• Of the 65 patients with OSAS, we found 10 with eyelid laxity, while the other 55 did not exhibit this feature. However, only one of the 10 patients with laxity showed signs of papillary conjunctivitis, so only one could fit the diagnosis of floppy eyelid syndrome. In the control group, 1 patient out of 39 had laxity of the upper eyelid, but no conjunctivitis (**Fig. 7**). The analysis showed a statistically significant association of eyelid laxity with OSAS, although only barely (Fisher’s exact test: p-value = 0.0495). As for FES, it could not be clearly associated with OSAS;

• We found that just 3.09% of the patients (2/ 65) had glaucoma in the OSAS group, and 10.26% (4/ 39) in the control (**Fig. 8**). The difference was not significant (Fischer’s exact test: p-value = 0.1940). We explained the high prevalence of glaucoma in the second group as the tendency of people to come to the ophthalmologist when they experience problems. However, the general prevalence for glaucoma in patients 40-80 years old was mentioned to be 3.54% [**12**] and that of POAG was 2.79% [**13**], and this was also not statistically different from what we found in our OSAS patients (p>0,99 and p=0.6968).

• The optic nerve head was in the vast majority normal, with a vertical cup/ disc ratio of 0,2-0,4 of symmetrical appearance. Only one patient exhibited a larger cup, of 0,5-0,6, associated with an IOP of 23-24 mmHg, and went on to receive a diagnosis of glaucoma, nevertheless not an advanced case according to the visual field and OCT. In addition, another patient had been already diagnosed with glaucoma and was following IOP lowering medication, accounting for the 2 cases found amid the 65 OSAS investigated patients.

• We further analyzed the OSAS patients with eyelid laxity, who had an AHI variability from a minimum of 17 to a maximum of 73 respiratory events/ hour. In patients without eyelid laxity, AHI varied from 6 to 82. In order to establish a correlation between AHI and eyelid laxity we statistically (unpaired T-test) analyzed and revealed that the difference between the groups was not significant (p=0,6185), although we can mention a slight tendency for a greater AHI in patients with laxity, as the medium AHI was 40 in these patients compared to 38 in the no-laxity group (**Fig. 9**).

**Fig. 10:**
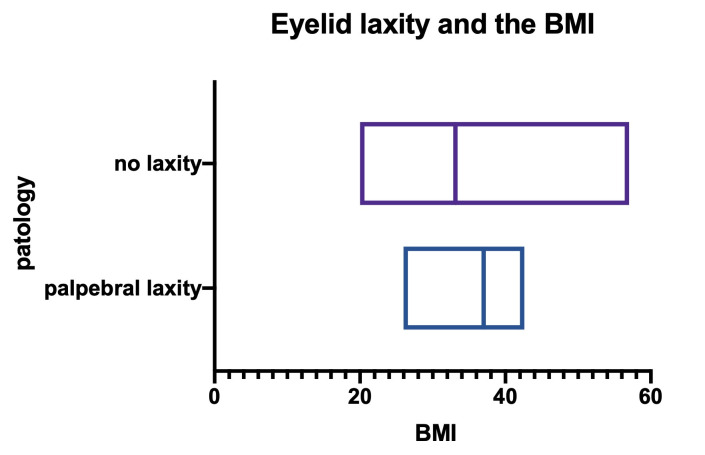
Body mass index compared between patients with and without palpebral laxity

**Fig. 11:**
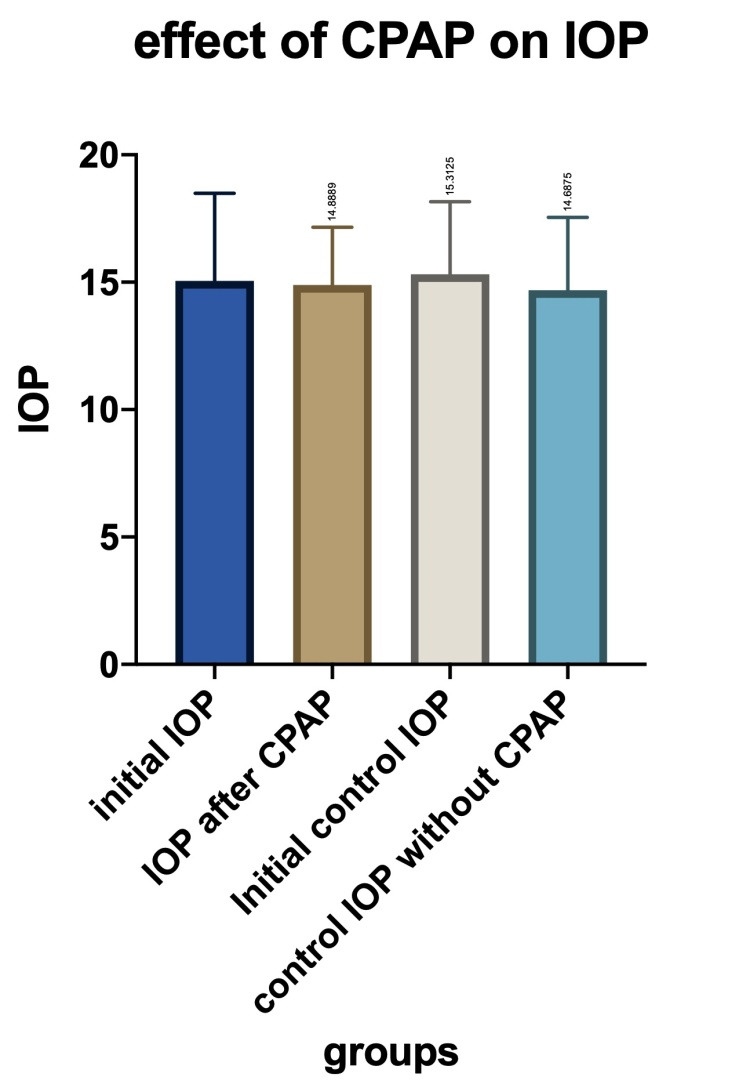
Initial IOP compared to IOP after CPAP treatment and to the IOP in the control group

**Fig. 12:**
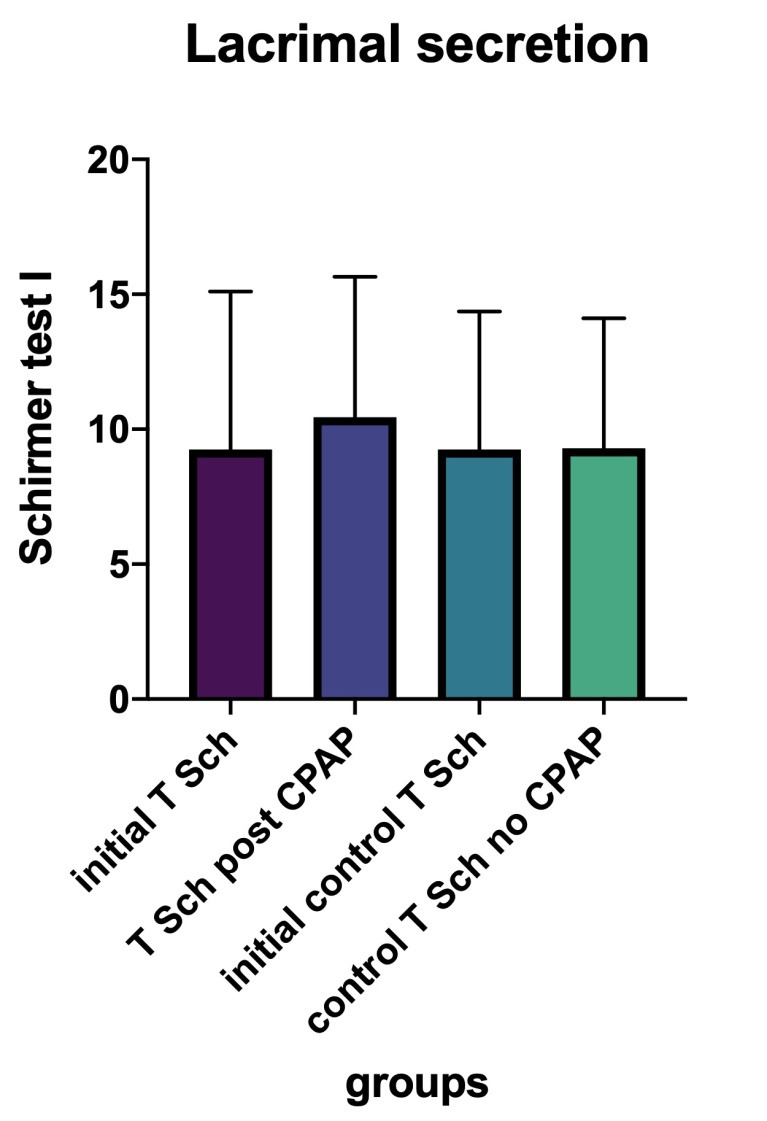
Initial Schirmer test values compared to after CPAP treatment and to control patients

• We analyzed 8 patients who manifested eyelid laxity versus 39 without this characteristic (**Fig. 10**). We noticed a tendency of the BMI (body mass index) to be higher in patients with eyelid laxity, but without statistical significance (p=0,4784). As a medium value, the palpebral laxity patients had a BMI of 35,19, while the other group had a BMI of 33,18 (r squared = 0,01123).

• Regarding the longitudinal part of the study, based on the analysis of 10 patients treated with CPAP versus 10 no-CPAP treated patients, we could draw the following conclusions: Initial medium ocular tension was 15.06 mmHg, and around 3 months later (2.5 - 4 months interval), the value dropped slightly to 14.89 mmHg (**Fig. 11**). The difference between initial and final values was very small, with no statistical significance (paired T-test: p-value = 0,8327);

• The further analysis of the 10 patients with SASO who underwent treatment by CPAP revealed the following: total lacrimal secretion (measured by Schirmer I test) was significantly improved following the average 3 months (2.5-4) of treatment (paired T-test, p-value = 0,0118). The medium at the beginning of treatment was 9.25 mm (5 min), compared to a 10.45 mm (at 5 min) value at the end of follow-up. The other 10 patients who were considered as a control group did not show the same improvement: their final values were not significantly different from the initial ones (paired T-test: p-value = 0,9420), with the initial medium value of 9.25 mm and the final one of 9.3 mm (**Fig. 12**);

## Discussion

According to studies, the prevalence of obstructive sleep apnea syndrome is estimated at 3-7% with certain groups of the population, like obese middle age men, bearing a higher risk [**14**]. OSAS is defined as AHI > 5/ h determined by PSG, associated with symptoms like excessive daytime sleepiness, loud snoring, etc. There are some inconsistencies in PSG determination of the disease, both between different labs and within the same lab. Reasons for these are the following: the threshold for oxyhemoglobin desaturation considered in the hypopnea definition might vary (3/ 4%), abbreviated monitorization, night variability, the “first night” effect, etc.

Some researchers present glaucoma as a frequent association of OSAS. In the present study, at least ophthalmoscopically, most patients did not exhibit glaucoma signs. As described above, only 2 patients were diagnosed and they had very light, interpretable lesions on OCT and on the automated (static) visual field. According to some studies, the ganglion cell complex (GCC) is affected in patients with OSAS. It was found to be thinner in the inferior and inferior nasal sector, in both eyes [**15**]. This may be regarded as a glaucoma sign, or an independent, non-related affliction.

Floppy eyelid syndrome frequently affects middle aged obese men, so it has a similar target population as OSAS. One study examined 8 patients with FES and turned out that all had OSAS. Opposingly, from 20 OSAS patients, only one had FES, and two other had early symptoms of this disease [**3**]. It seems that FES is a strong predictor of OSAS, so it is reasonable to assume that these patients might benefit from sleep studies. At present, the role of the ophthalmologist is to discover the patients and send them to the sleep specialist. One easy to miss ocular affliction might hide a devastating disease with repercussions on other organs of the body.

OSAS patients may exhibit eye surface problems like papillary conjunctivitis, corneal punctate epitheliopathy and recurrent corneal erosions [**2**,**16**-**18**]. These may be secondary to floppy eyelid syndrome or dry eye. According to the scientific literature, people with OSAS, regardless of the severity group, tend to exhibit significantly shorter tear break-up times (TBUT). In addition, significant positive correlations appeared between OSDI (ocular surface disease index) score, Schirmer test and both AHI and BMI. In one study, AHI and BMI increased, both TBUT and Schirmer values diminished [**19**]. The present research similarly found a strong correlation between OSAS severity and lacrimal function measured by Schirmer I test. Nevertheless, the OSDI did not seem particularly high and patients rarely complained of ocular symptoms. It is important to note that sleep deprivation itself seems to lower tear production and to cause dry eye in experimental models [**20**].

## Conclusions

Glaucoma does not seem to be associated with OSAS. IOP is similar in OSAS patients and in the general population. On the contrary, lacrimal secretion does seem to be diminished in OSAS patients.

FES is not frequent in OSAS but it has the potential to develop since there is an increased eyelid laxity. Night-time eversion of the abnormal lids can lead to symptomatic ocular surface irritation and papillary conjunctivitis.

Patients were followed after CPAP, and this treatment had an insignificant effect on IOP. However, CPAP treatment does improve lachrymal function. 

**Conflict of interest**

The authors declare no conflict of interest.

**Financial Disclosure**

None of the authors has any financial interest to disclosure.
